# NAD metabolism-related genes provide prognostic value and potential therapeutic insights for acute myeloid leukemia

**DOI:** 10.3389/fimmu.2024.1417398

**Published:** 2024-06-20

**Authors:** Yuncan Cao, Wenjing Shu, Peng Jin, Jianfeng Li, Hongming Zhu, Xinjie Chen, Yongmei Zhu, Xi Huang, Wenyan Cheng, Yang Shen

**Affiliations:** ^1^ Shanghai Institute of Hematology, State Key Laboratory of Medical Genomics, National Research Center for Translational Medicine at Shanghai, Ruijin Hospital Affiliated to Shanghai Jiao Tong University School of Medicine, Shanghai, China; ^2^ School of Life Sciences and Biotechnology, Shanghai Jiao Tong University, Shanghai, China; ^3^ Department of Critical Care Medicine, Renji Hospital, School of Medicine, Shanghai Jiaotong University, Shanghai, China

**Keywords:** acute myeloid leukemia, NAD metabolism, prognostic signature, immune microenvironment, targeted therapy

## Abstract

**Introduction:**

Acute myeloid leukemia (AML) is an aggressive blood cancer with high heterogeneity and poor prognosis. Although the metabolic reprogramming of nicotinamide adenine dinucleotide (NAD) has been reported to play a pivotal role in the pathogenesis of acute myeloid leukemia (AML), the prognostic value of NAD metabolism and its correlation with the immune microenvironment in AML remains unclear.

**Methods:**

We utilized our large-scale RNA-seq data on 655 patients with AML and the NAD metabolism-related genes to establish a prognostic NAD metabolism score based on the sparse regression analysis. The signature was validated across three independent datasets including a total of 1,215 AML patients. ssGSEA and ESTIMATE algorithms were employed to dissect the tumor immune microenvironment. *Ex vivo* drug screening and *in vitro* experimental validation were performed to identify potential therapeutic approaches for the high-risk patients. *In vitro* knockdown and functional experiments were employed to investigate the role of *SLC25A51*, a mitochondrial NAD+ transporter gene implicated in the signature.

**Results:**

An 8-gene NAD metabolism signature (NADM8) was generated and demonstrated a robust prognostic value in more than 1,800 patients with AML. High NADM8 score could efficiently discriminate AML patients with adverse clinical characteristics and genetic lesions and serve as an independent factor predicting a poor prognosis. Immune microenvironment analysis revealed significant enrichment of distinct tumor-infiltrating immune cells and activation of immune checkpoints in patients with high NADM8 scores, acting as a potential biomarker for immune response evaluation in AML. Furthermore, *ex vivo* drug screening and *in vitro* experimental validation in a panel of 9 AML cell lines demonstrated that the patients with high NADM8 scores were more sensitive to the PI3K inhibitor, GDC-0914. Finally, functional experiments also substantiated the critical pathogenic role of the *SLC25A51* in AML, which could be a promising therapeutic target.

**Conclusion:**

Our study demonstrated that NAD metabolism-related signature can facilitate risk stratification and prognosis prediction in AML and guide therapeutic decisions including both immunotherapy and targeted therapies.

## Introduction

Acute myeloid leukemia (AML) is an aggressive hematopoietic malignancy characterized by high heterogeneity and poor prognosis (1). Despite advances in therapeutic approaches, the global 5-year survival rate remains significantly lower at approximately 30% (2). Refractory and relapsed AML persist as the major obstacles within the current treatment paradigms (3). Cytogenetic and molecular abnormalities are crucial for the evaluation of prognosis and assignment of therapeutic strategies. However, under current risk stratification criteria such as the European Leukemia Net (ELN) recommendations (4), patients within each risk group still demonstrate substantial heterogeneity in terms of clinical outcomes and biological abnormalities.

With the widespread adoption of next-generation sequencing (NGS) technologies, it becomes feasible to comprehensively explore the genomic and transcriptomic profiles, which may largely promote the precise classification and prognostic evaluation of AML patients (5–7). To date, numerous genome- and transcriptome-based AML prognostic models have been developed, including the 17-gene stemness score (LSC17) defined by stem cell subsets (8), the 16-gene AML fitness (AFG16) from large-scale CRISPR-Cas9 screening (9), and the GENE4 generated by capturing intratumor heterogeneity of AML (10), indicating that the gene transcriptional data could capture the heterogeneity of AML patients and largely refine the traditional risk assignment system. In this context, integrating multi-omics data offers insights to identify novel molecular markers with prognostic and therapeutic value in AML, enabling more precise therapy and refined stratification, which represents a major area of future research.

Metabolic reprogramming is a prototypical hallmark of tumor development, which can not only serve as a direct manifestation of the functional status of tumors but may also play a critical role in the pathogenesis of various cancers (11). The rewiring of multiple essential metabolic pathways including glycolysis, lipid metabolism and glyceraldehyde-3-phosphate dehydrogenase (GAPDH) has been discovered to be associated with a more aggressive leukemic phenotype and drug resistance in AML (12–14). Aberrations in key metabolic enzymes greatly influence the levels of metabolites, potentially affecting epigenetic processes including DNA methylation, histone modification, and chromatin remodeling. For instance, in AML, the oncometabolite 2-hydroxyglutarate (2-HG) generated by *IDH1/2* mutations competes with α-ketoglutarate (α-KG) for binding, leading to extensive methylation of DNA and histones, disrupting crucial hematopoietic signaling pathways and facilitating the onset of AML (15). Upregulation of the gene *SLC2A5*, encoding the fructose transporter protein GLUT5, results in increased fructose uptake, compensating for the relative fructose deficiency of tumor cells, thereby promoting leukemia cell survival (16). Currently, inhibitors targeting aberrant key metabolic enzymes such as IDH, GPX4, and NAMPT have shown promising anti-tumor effects (17, 18).

Nicotinamide adenine dinucleotide (NAD) is a key mediator participating in a variety of biological processes, including energy metabolism, redox reactions, DNA repair, immune responses, and protein acetylation. Besides, NAD serves as the substrate for numerous enzymes such as sirtuins and poly ADP-ribose polymerases (PARPs) (19). Dysregulation of NAD metabolism is essential in the initiation and progression of various cancers, which may provide specific adaptations to support the growth and survival of malignant cells (20–22). It has been reported that the dysregulated NAD metabolism enables leukemia stem cells (LSCs) to evade apoptosis, leading to their resistance to multiple drugs including traditional chemotherapy and BCL2 inhibitor venetoclax (23). Moreover, the inhibition of nicotinamide phosphoribosyltransferase (NAMPT), a rate-limiting enzyme in the NAD metabolism, demonstrated selective eradication of LSCs in relapsed AML patients (24). These results underscore the critical pathogenic role of NAD metabolism. However, the prognostic significance of NAD metabolism-related genes and their potential as therapeutic targets in AML have not been fully elucidated.

Therefore, our study focused on delineating the NAD metabolic profiles in AML from the perspective of transcriptomics. After systematically profiling the NAD metabolism-related genes in our large cohort of AML patients, we innovatively developed an 8-gene signature which demonstrated a robust prognostic value across diverse independent validation datasets. The signature could exquisitely discriminate AML patients with aberrant NAD metabolism characteristics and poor prognosis. More importantly, further immune microenvironment investigation and drug sensitivity exploration provided therapeutic insights for high-risk AML patients, lending support to the clinical implementation of this prognostic model.

## Materials and methods

### Data availability

The training cohort comprised 655 newly diagnosed AML patients from three large centers, 442 of these patients were from Shanghai Institute of Hematology (SIH), 110 from Jiangsu Institute of Hematology (JIH), and 103 from Zhejiang Institute of Hematology (ZIH). All bone marrow mononuclear cell (BMMC) samples from 655 AML patients were collected at diagnosis. Anonymized RNA sequencing data have been deposited in the Genome Sequence Archive for Humans (GSA-Human, https://ngdc.cncb.ac.cn/gsa-human) (HRA002693). The detailed treatment information of these patients is elaborated in the **Supplementary Methods** and **Material**. The diagnosis and classification of AML patients were conducted based on the 2022 World Health Organization criteria. This study was approved by the three participating centers. All patients had given informed consent for both treatment and cryopreservation of BMMC samples according to the Declaration of Helsinki.

The validation cohorts were obtained from the TCGA (https://portal.gdc.cancer.gov/), BeatAML (http://www.vizome.org/), and HOVON cohort (retrieved from Array Express with Dataset ID: E-MTAB-3444). Only treatment-naive adult patients were included, and individuals without available survival information were excluded.

### Acquisition of NAD+ metabolism-related genesets

The NAD+ metabolism-related genes were obtained from the pathways from Molecular Signatures Database (MSigDB) (25), including REACTOME_NICOTINATE_METABOLISM(R-HAS-196807) from Reactome database, KEGG_NICOTINATE_AND_NICOTINAMIDE_METABOLISM (has00760) from KEGG database, and GOBP_DE_NOVO_NAD_BIOSYNTHETIC_PROCESS, GOBP_NAD_TRANSPORT from GO database. After integrating the genes in these pathways, a total of seventy-seven genes were selected. Detailed information on the NAD+ metabolism-related genes is provided in **Supplementary Table S1**.

### Development and validation of AML prognostic model

The least absolute shrinkage selector operator (LASSO) regression algorithm was employed in the *glmnet* R package to select the fitness genes, which ultimately generated eight genes most pertinent to prognosis. Then, the 8 genes were incorporated into the multivariate Cox proportional-hazards (CPH) regression and established the NADM8 model. The NADM8 risk score was calculated using the formula: NADM8 score = Σ (β*i* × expression level of gene*i*), where βi was the coefficient generated by multivariable CPH regression analysis. Furthermore, each patient was allocated a risk score according to the formula and subsequently divided into high-risk and low-risk subgroups according to the median cut-off risk score. Kaplan-Meier survival analysis was employed to assess the prognostic implications of the NADM8 model using the *survival* and *survminer* R package. The time-dependent ROC curves and area under time-dependent ROC curve (AUC) were performed using the *timeROC* package.

### Gene set variation analysis and pathway survival analysis

We systematically collected the NAD metabolism-related pathways from the “msigdb.v2023.1.Hs.symbols.gmt” in MSigDB database and conducted Gene Set Variation Analysis (GSVA) utilizing the *GSVA* R package to calculate a score of each pathway for the patients. Then, survival analysis was performed using the univariate CPH regression analysis and Kaplan-Meier curves to explore the prognostic value of these pathways.

### Tumor immune microenvironment estimation

We evaluated the abundances of the 28 immune cells infiltrating the AML microenvironment based on ssGSEA R package and compared the differences of the immune cell infiltration between the NADM8^low^ and NADM8^high^ groups. The immune scores of different risk groups were calculated through the *estimate* R package. Then, we computed the correlation coefficients between the selected immune cells and the 8 NAD genes of the model and demonstrated by a heatmap.

### Drug sensitivity assays

A panel of AML cell lines (HL-60, U937, OCI-AML3, Kasumi-1, ME1, NB4, MOLM13, OCI-AML2, K562) were exposed to varying concentrations of GDC-0941 (Selleckchem, S2065) in 96-well plates at a density of 2 × 10^4^ cells per well. After incubation for 48h, cell viability was assessed using the Cell Counting Kit-8 (CCK-8) assays. CCK-8 solution was added to 96 well plates and further incubated with the cells at 37 °C, 5% CO2 for 4 h. Then, the absorbance of the samples was measured at 450 nm. The RNA sequencing (RNA-seq) data of AML cell lines were downloaded from the Cancer Cell Line Encyclopedia (CCLE) (https://sites.broadinstitute.org/ccle/datasets) and the risk scores were calculated according to the NADM8 equation. Pearson’s regression analysis was conducted to examine the correlation between risk scores and IC50 values.

In order to validate the therapeutic efficacy of the PI3K inhibitor GDC-0941 in primary AML samples, we randomly selected BMMC samples from three NADM8^high^ and three NADM8^low^ AML patients in our RJAML cohort. BMMCs were plated in a 96-well plate at the density of 1×10^4^ cells per well and exposed to GDC-0941 at gradient concentrations. After incubation for 48h, cell viability was evaluated by CCK-8, and the IC50 value was calculated.

### Functional experiments in AML cell lines

Knockdown of the *SLC25A51* gene was constructed by virtue of a pLVX plasmid carrying shRNA, which along with psPAX2 and pMD2.G package plasmids were co-transfected into HEK293T cells using Lipofectamine 2000. The supernatant was collected at 24h and 48h post-transfection to obtain the viral particles. Subsequently, THP-1 and U937 cells were transfected with lentiviral supernatant in the presence of 8 μg/μl polybrene, and subjected to centrifugation at 1200g for 90 minutes at 30°C–32°C. After 48 hours, GFP positivity was assessed by flow cytometry to determine transduction efficiency. RNA was extracted to evaluate the knockdown efficiency through quantitative methods. At 72 hours post-transduction, flow cytometry was employed to assess the apoptosis (Invitrogen™ eBioscience™ Annexin V Apoptosis Detection Kits) and cell cycle distribution (Cell Cycle Staining Kit, Multi Science). Cell proliferation was monitored for four consecutive days using the CCK-8 assay (Cell Counting Kit-8, DOJINDO).

### Statistical analyses

For the statistical significance estimation of clinical characteristics and outcome, the Student’s t-test was applied for normally distributed quantitative data, the Wilcoxon test for non-normally distributed continuous variables, and the Fisher’s exact test for categorical data. The Cochran-Mantel-Haenszel (CMH) test was employed to assess unidirectional ordered contingency tables. The mutational profile was analyzed via the *maftools* package. The mutation waterfall plot was drawn using the R package *complexheatmap*. Utilizing the *survival*, *regplot* and *rms* R packages, we constructed a nomogram and generated the calibration curve. The GO and KEGG analysis were performed by R package *clusterProfler* with the reference gene sets from “c5.all.v2023.2.Hs.symbols.gmt” and “c2.cp.kegg.v7.5.1.symbols”, which were downloaded from MSigDB. All statistical analyses in this study were conducted by R 4.3.1, and bilateral *P* value<0.05 was determined as statistically significant.

## Results

### Construction of an AML prognostic model based on NAD metabolism-related genes

**Figure 1:**
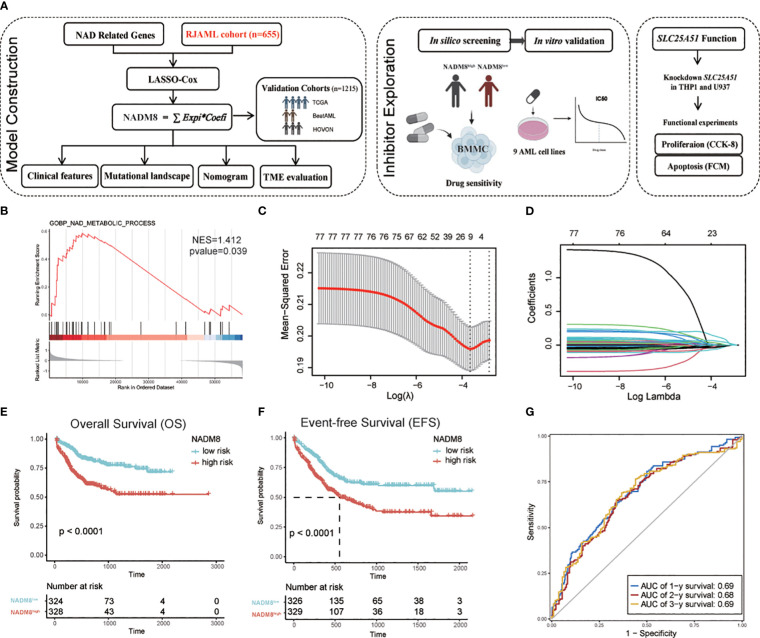
Development of the NAD metabolism-related AML prognostic model **(A)** Flow diagram of this research. **(B)** GSEA shows the enrichment of “GOBP_NAD_METABOLIC_PROCESS” in the patients dead within two years after diagnosis compared to those who survived in the TCGA cohort (*P*=0.039, NES=1.412). **(C, D)** The LASSO regression algorithm was employed to identify the fitness genes. 77 NAD metabolism-related genes were initially inputted and 8 genes were selected for further model construction **(C)** The partial likelihood deviance in the LASSO analysis. **(D)** LASSO coefficient profiles of the eight screened NAD metabolism-related genes. **(E, F)** Kaplan-Meier estimates of overall survival (OS) **(G)** and event-free survival (EFS) according to the NADM8 score in the training cohort. **(G)** Time-dependent ROC curves show the 1-year, 2-year, and 3-year prediction accuracy of the NADM8 score.

The workflow showed the processes of developing a prognostic signature associated with NAD metabolism in AML (**Figure 1A**). Ahead of this, we systematically deciphered the global dysregulation of NAD metabolism in AML. We firstly collected the NAD metabolism-related pathways from the MSigDB database and conducted Gene Set Enrichment Analysis (GSEA) based on the TCGA-LAML cohort. We found that the NAD metabolism signaling was significantly enriched in patients who succumbed within 2 years compared to those who survived (**Figure 1B**, p=0.039, NES=1.412). We also employed the GSVA algorithm to quantify the activity of NAD metabolism-related pathways in individual patient samples. Further univariate CPH regression analysis using the GSVA calculated activity score revealed that NAD metabolism-related pathways were significantly associated with a poor prognosis (**Supplementary Figures S1A–D**, Hazard Ratio>1, *P*<0.05). Then we aimed to construct a transcriptome-based prognostic model to precisely identify high-risk patients with dysregulated NAD metabolism.

A set of 77 NAD metabolism-related genes were obtained by integrating these pathways (**Supplementary Table S1**). Univariate CPH regression analysis was performed for each gene in our cohort of 655 newly diagnosed AML patients (**Supplementary Table S2**). We then employed the LASSO regression machine learning algorithm to perform an unbiased shrinkage of the 77 genes, which yielded 8 genes with the most prognostic relevance (**Figures 1C, D**). These genes were then incorporated into a multivariable Cox model, termed the 8-gene NAD metabolism score (NADM8), which is calculated as: NADM8 = NT5M*(-0.1451) + NMNAT2*(0.9138) + SIRT2*(0.1724) + ACMSD*(-0.5692) + HAAO*(-0.1707) + NUDT13*(-0.3434) + SARM1*(0.5218) + SLC25A51*(0.5287).

We assigned a risk score to each patient according to the NADM8 model and stratified the 655 patients into high-risk (NADM8^high^) and low-risk (NADM8^low^) groups by the median value as the cutoff. Patients with higher NADM8 risk scores exhibited significantly poorer overall survival (OS) and event-free survival (EFS) compared to those with lower risk scores (**Figures 1E, F**). In addition, the area under the curve (AUC) of the time-dependent receiver operating characteristic (ROC) curve at the 1-year, 2-year and 3-year overall survival was 0.69, 0.68 and 0.69, respectively, indicating the prognostic significance of the NADM8 signature (**Figure 1G**).

### Comparison of clinical features and molecular landscapes between NADM8^high^ and NADM8^low^ patients

Comparison of clinical characteristics of patients in the two groups showed that patients with high NADM8 scores were older (51.00 vs 46.00, *P*<0.001) and more frequently classified into the intermediate and adverse European LeukemiaNet (ELN) risk groups (Favorable 14.3% vs 45.1%, Intermediate 34.7% vs 22.4%, Adverse 45.9% vs 23.9%, *P*<0.001). Besides, patients with high NADM8 scores harbored more unfavorable cytogenetic aberrations such as complex karyotypes (11.2% vs 5.2%, *P*=0.004) and monosomal karyotypes (10.9% vs 5.8%, *P*=0.017). As for the French-American-British (FAB) classification, high-risk patients were significantly enriched in the M5 subtype, also termed acute monocytic leukemia (34% vs 16.3%, *P*<0.001) (**Table 1**).

**Table 1:** Clinical characteristics in the development cohort.

Characteristic	Overall (n=655)	NADM8^high^ (n=329)	NADM8^low^ (n=326)	P-value
Age (median [IQR])	48.00 [34.00, 60.00]	51.00 [37.00, 63.00]	46.00 [33.00, 58.00]	**<0.001 #**
WBC (median [IQR])	12.40 [3.52, 43.95]	13.06 [3.06, 42.73]	12.33 [3.95, 46.04]	0.456 #
BM Blast (median [IQR])	68.00 [45.00, 84.00]	66.00 [38.00, 83.00]	69.60 [52.00, 84.00]	**0.012 #**
HGB (median [IQR])	86.00 [68.00, 106.50]	83.00 [67.00, 102.00]	90.50 [68.00, 109.75]	**0.009 #**
PLT (median [IQR])	40.00 [23.00, 78.00]	52.00 [29.00, 90.00]	33.00 [20.00, 61.00]	**<0.001 #**
**Gender, n (%)**				0.085 $
Female	316 (48.2)	170 (51.7)	146 (44.8)	
Male	339 (51.8)	159 (48.3)	180 (55.2)	
**ELN2022, n (%)**				**<0.001 &**
Favorable	194 (29.6)	47 (14.3)	147 (45.1)	
Intermediate	187 (28.5)	114 (34.7)	73 (22.4)	
Adverse	229 (35.0)	151 (45.9)	78 (23.9)	
**FAB subtype, n (%)**				
M0	2 (0.3)	2 (0.6)	0 (0)	/
M1	33 (5.0)	10 (3)	23 (7.1)	**0.031 $**
M2	136 (20.8)	51 (15.5)	85 (26)	**0.008 $**
M3	56 (8.5)	20 (6.1)	36 (11)	**0.050 $**
M4	189 (28.9)	81 (24.6)	108 (33.1)	0.083 $
M5	165 (25.2)	112 (34)	53 (16.3)	**<0.001 $**
M6	1 (0.2)	0 (0)	1 (0.3)	/
M7	1 (0.2)	1 (0.3)	0 (0)	/
AML, NOS	72 (11)	52 (15.8)	20 (6.1)	/
***FLT3-*ITD, n (%)**				**<0.001 $**
Wild type	526 (80.3)	243 (73.9)	283 (86.8)	
Mutation	169 (25.8)	86 (26.1)	43 (13.1)	
***RUNX1::RUNX1T1*, n (%)**				**<0.001 $**
Negative	602 (91.9)	317 (96.4)	285 (87.4)	
Positive	53 (8.1)	12 (3.6)	41 (12.6)	
***CBFβ::MYH11*, n (%)**				**<0.001 $**
Negative	603 (92.1)	322 (97.9)	281 (86.2)	
Positive	52 (7.9)	7 (2.1)	45 (13.8)	
***KMT2A-rearranged, n (%)***				
Negative	615 (93.9)	301 (91.5)	314 (96.3)	**0.014 $**
Positive	40 (6.1)	28 (8.5)	12 (3.7)	
**Complex karyotype, n (%)**				**0.004 $**
Negative	580 (88.5)	279 (84.8)	301 (92.3)	
Positive	54 (8.2)	37 (11.2)	17 (5.2)	
**Monosomal karyotype, n (%)**				**0.017 $**
Negative	579 (88.4)	280 (85.1)	299 (91.7)	
Positive	55 (8.4)	36 (10.9)	19 (5.8)	
**Chemotherapy responses, n (%)**				**<0.001 $**
Response	251 (38.3)	111 (33.7)	140 (42.9)	
Non-response	84 (12.8)	58 (17.6)	26 (8.0)	

# P value calculated using the Wilcoxon rank-sum test.

$ P value calculated using the Fisher’s exact test.

& P value calculated by Cochran-Mantel-Haenszel Statistics (CMH) test.

P value less than 0.05 is shown in bold.

**Figure 2:**
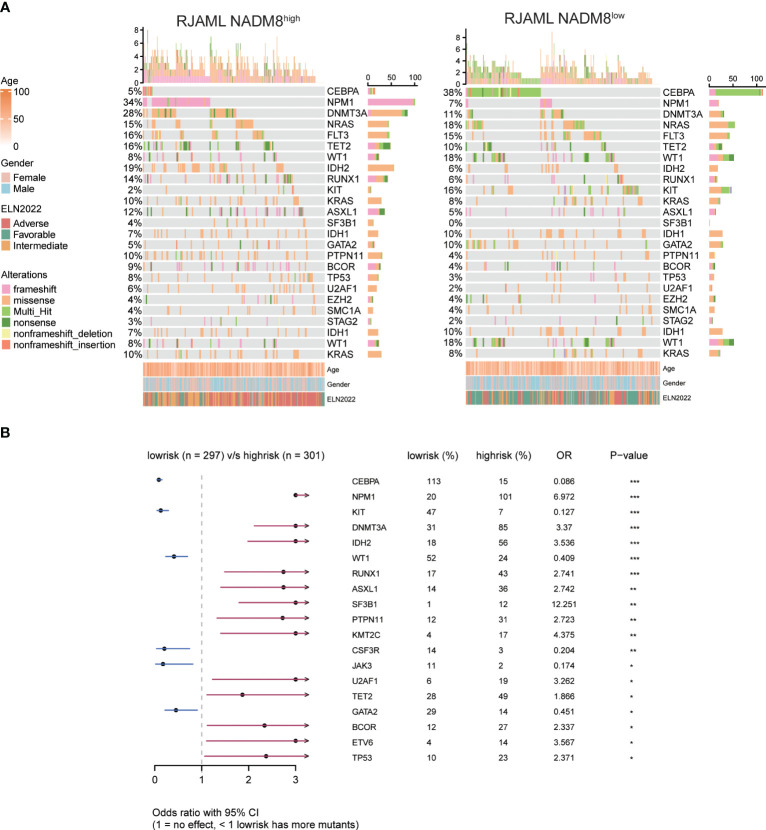
Comparison of mutational landscape and clinical features between NADM8^high^ and NADM8^low^ patients **(A)** Heatmap shows the clinical information and somatic mutations between NADM8^high^ and NADM8^low^ patient groups. **(B)** Forest plot manifests genetic mutations that occur at significantly different frequencies between NADM8^high^ and NADM8^low^ groups.

To obtain a more comprehensive insight into the mutational landscape associated with the NADM8 score, the frequency of common genetic lesions was compared between NADM8^high^ and NADM8^low^ groups (**Figure 2A**). Notably, *KMT2A* rearrangements, *FLT3* internal tandem duplication (*FLT3*-ITD), and mutations of *NPM1, DNMT3A, IDH2, RUNX1, ASXL1, SF3B1, PTPN11, KMT2C, U2AF1, TET2, BCOR1, ETV6, TP53* genes were more frequently observed in the NADM8^high^ group. In contrast, patients in the NADM8^low^ group harbored more bZIP *CEBPA* mutations and core-binding factor (CBF) fusions, including *RUNX1::RUNX1T1* and *CBFB::MYH11* (**Figure 2B**).

Differentially expressed genes (DEGs) analysis identified 95 upregulated genes and 66 downregulated genes in the NADM8^high^ group in comparison with the NADM8^low^ group (**Supplementary Figures S2A, B**; **Supplementary Table S5**). Remarkably, *HOX* family genes, including *HOXA9, HOXA10, HOXA6*, and *HOXA5* involved in the development of AML, were notably enriched in the high-risk group.

### Independent prognostic value assessment of the NADM8 model and nomogram establishment

**Figure 3:**
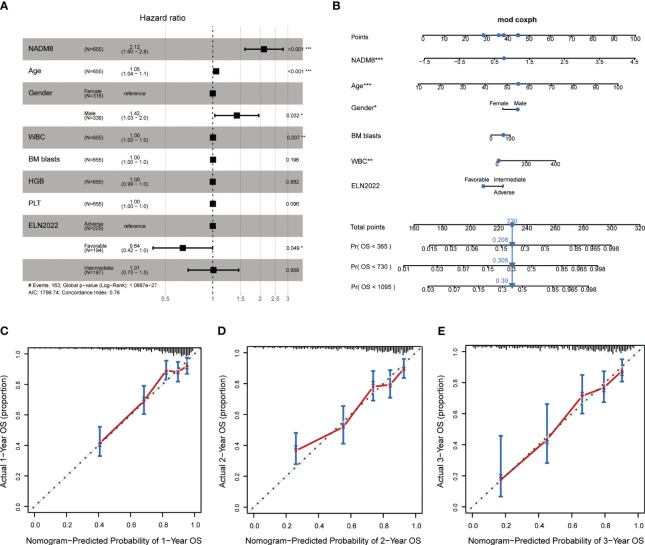
Establishment of a Nomogram integrating NADM8 score and common clinical parameters. **(A)** Multivariate Cox proportional-hazards regression analysis indicates that NADM8 score is prognostically independent of common clinical factors (p<0.001) **(B)** A nomogram integrating NADM8 and clinical parameters such as WBC and ELN risk for the 1-year, 2-year, and 3-year overall survival probability prediction. **(C–E)** Calibration curves show the predicted and actual 1-year, 2-year, and 3-year survival probabilities in the training cohort. WBC, white blood cell count; ELN, European LeukaemiaNet.

In Multivariate CPH models, the NADM8 risk score retained significant prognostic value independent of common clinical predictors including age, gender, white blood cell (WBC) count, Hemoglobin (HGB), platelet (PLT), bone marrow (BM) blasts, and ELN risk stratification (**Figure 3A**). Furthermore, the integration of NADM8 could raise the Concordance index (C-index) of the CPH model in our AML cohort from 0.74 to 0.76 (**Supplementary Table S6**). These results indicate that the NADM8 signature could facilitate prognostic assessment as an independent risk factor.

In pursuit of a clinically applicable and convenient tool to predict the survival of AML patients, we constructed a nomogram model based on the multivariate CPH model, integrating both clinical covariates and the NADM8 risk score (**Figure 3B**). The 1-, 2- and 3-year calibration curves exhibited a high consistency between the actual and predicted probabilities of OS, suggesting that the nomogram possesses excellent concordance in predicting the prognosis of AML patients (**Figures 3C–E**).

### The NADM8 model is robustly associated with overall survival in multiple independent AML cohorts

**Figure 4:**
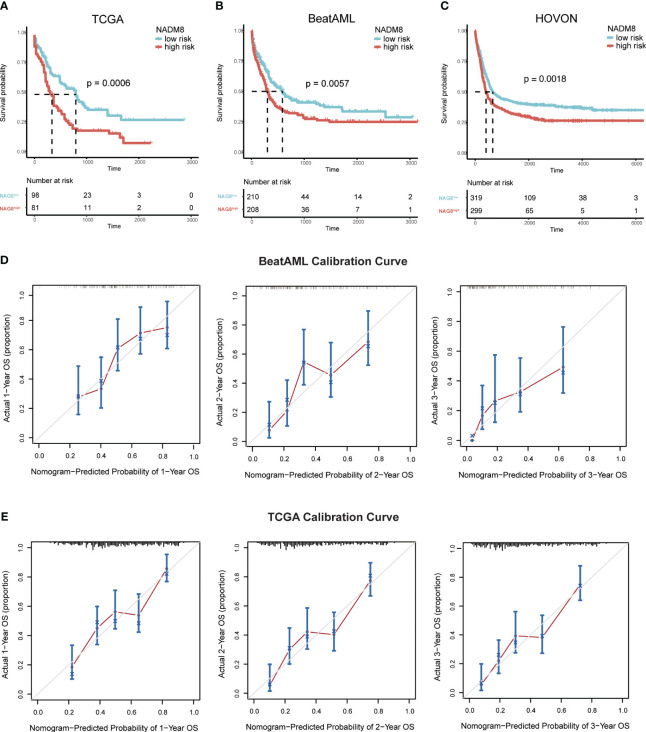
Validation of the prognostic value of the NADM8 score across multiple independent datasets. **(A-C)** Kaplan-Meier estimates of OS according to the NADM8 score in the BeatAML cohort **(A)**, TCGA cohort **(B)**, and HOVON cohort **(C)**. **(D, E)** Calibration curves of the nomogram model show the predicted and actual 1-year, 2-year, and 3-year survival probabilities in the BeatAML cohort **(D)** and TCGA cohort **(E)**.

Subsequently, we sought to comprehensively assess the prognostic performance of NADM8 model across several independent validation cohorts, including the TCGA (n=179), BeatAML (n=418), and HOVON (n=618) datasets. Of note, the NADM8 score was consistently correlated with a significantly worse OS in different AML cohorts (TCGA: *P*=0.0006, BeatAML: *P*=0.0057, HOVON: *P*=0.0018) (**Figures 4A–C**). Additionally, the calibration curves for the 1-, 2- and 3-year OS rates demonstrated a high consistency between the actual and predicted probabilities (**Figures 4D, E**). These results highlight the effectiveness of the NADM8 model as a reliable prognostic tool.

Next, we examined the differences between the two risk groups within the validation datasets (**Supplementary Tables S3**, **4**; **Supplementary Figures S3A–D**). Within both the TCGA and BeatAML cohorts, the NADM8^high^ group was associated with a significantly older age and higher proportion of ELN intermediate- and high-risk patients, as well as a higher frequency of *TP53* mutations, complex karyotype, and FAB-M5 subtype. Additionally, in the BeatAML cohort, the frequency of *SF3B1, RUNX1, FLT3-ITD* mutations and *KMT2A* rearrangements was higher in the NADM8^high^ group, while *CEBPA* mutations and *CBFB::MYH11* were relatively scarce. Collectively, the discrepancies between the two groups were consistent with the results in the development cohort.

### NADM8 outperforms other transcriptome-based risk models

To further ascertain the strength and robustness of our model, we compared the NADM8 score with previously reported transcriptome-based prognostic models, such as the 17-gene stemness score (LSC17) (8) and the 16-gene AML fitness (AFG16) (9). When adjusting for common clinical covariates, the NADM8 model exhibited generally superior performance in multiple datasets (TCGA, BeatAML, and HOVON cohorts), as reflected in the most significant *P* value in all multivariate Cox models, whereas other models only retained significant prognostic value in a subset of these cohorts. On the other hand, the CPH model showed a slightly higher Harrell C-index when integrating the NADM8 score across the three cohorts (BeatAML, NADM8: 0.71, LSC17: 0.70, AFG16: 0.71, and TCGA, NADM8: 0.74, LSC17: 0.73, AFG16: 0.73) (**Supplementary Tables S7**, **8**). Overall, these results suggest that the NADM8 score is independent of common clinical factors and could be a robust prognostic predictor of AML survival.

### Assessment of tumor immune infiltration landscapes between the two risk groups

**Figure 5:**
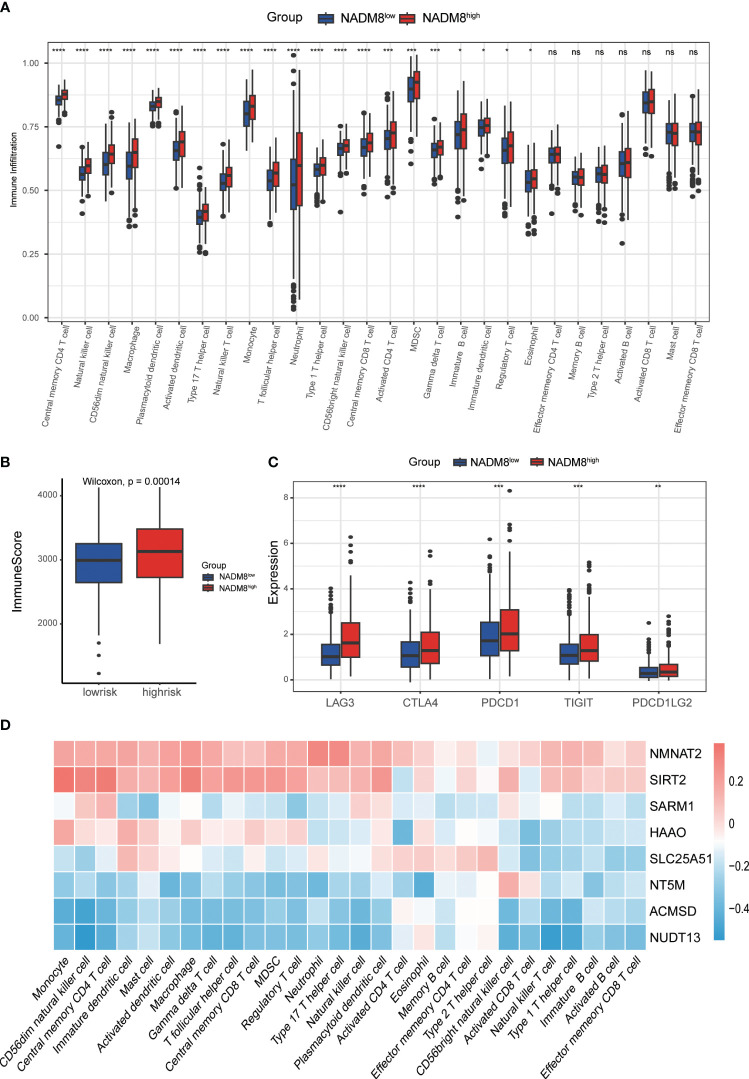
Correlations of immune microenvironment features with the NADM8 signature. **(A)** The differential infiltration of multiple immune cells between NADM8^high^ and NADM8^low^ samples. **(B)** Comparison of immune activity between NADM8^high^ and NADM8^low^ samples via the ESTIMATE algorithm. **(C)** The expression levels of immune checkpoint molecules between the NADM8^high^ and NADM8^low^ groups **(D)** The correlation between the 8 NAD-related genes and infiltrated immune cells in the TME. TME, tumor microenvironment. * means p < 0.05; ** means p < 0.01; *** means p < 0.001; **** means p < 0.0001; ns means no significance.

To investigate the impact of the NADM8 score on the immune microenvironment in AML, we examined the abundance of 28 types of immune cells between the two NADM8 risk groups. The results demonstrate that higher NADM8 scores were significantly correlated with increased infiltration of various immune cells, including central memory CD4 T cell, natural killer cell, CD56^dim^ natural killer cell, macrophage, plasmacytoid dendritic cell, activated dendritic cell, type 17 T helper cell, natural killer T cell, monocyte, T follicular helper cell, neutrophil, type 1 T helper cell, CD56bright natural killer cell, central memory CD8 T cell, activated CD4 T cell, myeloid-derived suppressor cells (MDSCs), Gamma delta T cell, immature B cell, immature dendritic cell, regulatory T cell and eosinophil (P < 0.05) (**Figure 5A**). In addition, NADM8^high^ patients have a higher immune score calculated through the “estimate” algorithm (**Figure 5B**).

Immune checkpoint molecules are pivotal targets for immunotherapy and crucial indicators of the efficacy of immunotherapy. The expression levels of common immune checkpoints, including *CTLA4*, *PDCD1*, *PDCD1LG2*, *LAG3* and *TIGIT* were significantly higher in high-risk individuals than in low-risk patients (**Figure 5C**). The heatmap showed the correlation between the 8 NAD-related genes of the model and the level of immune cell infiltration (**Figure 5D**). Overall, these findings underscore the significance of the NADM8 score as a potential biomarker for immune response evaluation in AML, providing insights for the development of novel therapeutic approaches.

### High NADM8 score indicates the potential therapeutic efficacy of specific small-molecule inhibitors

**Figure 6:**
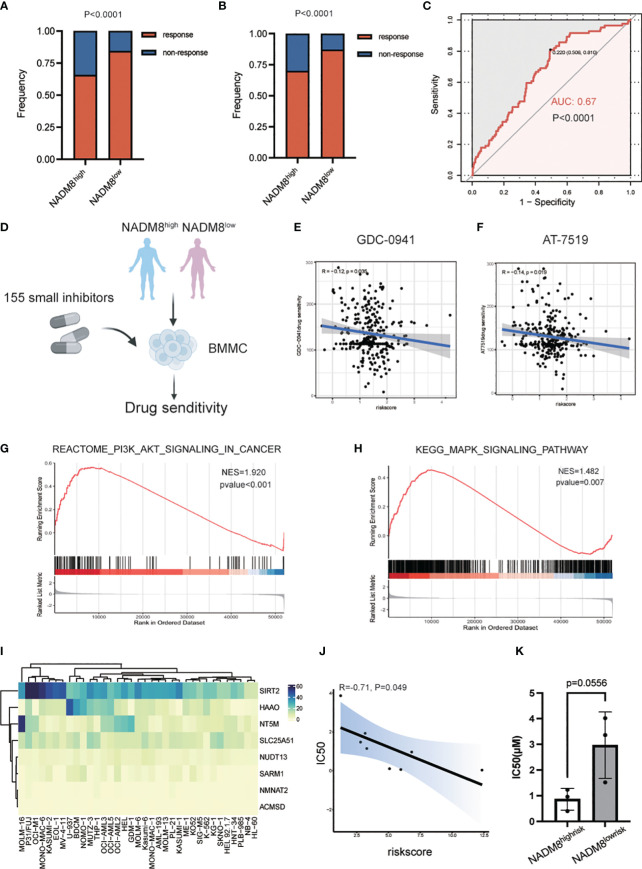
Exploration of effective specific small-molecule inhibitors for the NADM8^high^ patients. **(A)** Bar plot shows the distribution of chemotherapy response rate between the NADM8^high^ and NADM8^low^ patients in all the RJAML cohort and **(B)** the patients who received the “3 + 7”-based regimens as initial induction in the development cohort. **(C)** ROC curve indicates the prediction power of NADM8 for chemotherapy resistance in the univariate logistic regression model (AUC=0.67, *P*<0.001). **(D)** Illustrative diagram of *ex vivo* drug sensitivity data in AML patients from the BeatAML cohort. The freshly isolated mononuclear cells from patients were exposed to 155 small-molecule inhibitors at different concentrations and the drug sensitivity of these patient-derived cells was further determined. **(E, F)** Pearson’s correlation analysis identifies the potential drugs, GDC-0941 and AT-7519, that exert therapeutic effect in NADM8^high^ patients. Significant inhibitors were determined using the threshold of p-value < 0.05. **(G, H)** GSEA shows the enrichment of PI3K-AKT-mTOR signaling and MAPK signaling pathway in NADM8^high^ patients. **(I)** Heatmap depicts the expression level of the 8 NAD-related genes in AML cell lines. **(J)** The Pearson’s correlation plot shows that the NADM8 scores of the 9 AML cell lines are negatively associated with the IC50 score of GDC-0941 generated from *in vitro* drug sensitivity assays. **(K)** Boxplot showing IC50(μM) between samples of NADM8^high^ and NADM8^low^ patients against GDC-0941.

Exploration of the treatment responses after two courses of induction chemotherapy of 335 AML patients in our training cohort manifested that the complete remission (CR) rate of the NADM8^high^ group was significantly lower than that in the NADM8^low^ group (*P*<0.001) (**Figures 6A, B**; **Supplementary Table S9**), indicating a less sensitivity of high-risk patients to conventional chemotherapy. In addition, in the univariate logistic regression model, the NADM8 score demonstrated predictive power for chemotherapy resistance (**Figure 6C**, AUC=0.67, *P*<0.001).

Therefore, there is an urgent need to identify novel potential agents that could serve as alternative therapeutic options for these high-risk AML patients. To meet this clinical exigency, we exploited the *ex vivo* drug sensitivity profiles of AML samples to a panel of small-molecule inhibitors from the BeatAML cohort (26) (**Figure 6D**). Pearson’s correlation analysis was applied between the area under the dose-response curve (AUC) of each drug and the NADM8 score of AML patients. Two drugs, GDC-0941 (Pictilisib) and AT7519, were identified to be potential effective therapeutic agents for high-risk patients, whose AUC were negatively correlated with the risk score (both *P*<0.05) (**Figures 6E, F**).

GDC-0941, a pan-phosphatidylinositol-3-kinase (PI3K) inhibitor, has been incorporated into clinical trials in combination with paclitaxel for metastatic breast cancer (NCT01740336, NCT00960960) (27, 28), and with either paclitaxel, carboplatin, or cisplatin for advanced non-small-cell lung cancer (29). Besides, GDC-0941 has been validated to induce growth arrest and apoptosis of AML cells. AT7519, a novel multi-cyclin-dependent kinase inhibitor, could induce cell apoptosis in multiple myeloma (30) and glioblastoma (31). In concordance with this, the PI3K-AKT-mTOR and MAPK signaling pathways were significantly upregulated in NADM8^high^ patients (**Figures 6G, H**). Accordingly, we hypothesize that a high NADM8 score probably reflects certain biological features of AML blasts, which might confer greater sensitivity to these two targeted therapies. Overall, these findings suggest that the NADM8 score could be employed to facilitate the effective use of drug candidates like GDC-0941 and AT7519 for AML patients who may poorly respond to conventional induction chemotherapy.

To further validate the therapeutic efficacy of the predicted drugs, we profiled the sensitivity of 9 AML cell lines to PI3K inhibitor GDC-0941 through *in vitro* experiment. (**Supplementary Figures S4A–I**). Meanwhile, the NADM8 score of each AML cell line was calculated using the RNA-seq data from the Cancer Cell Line Encyclopedia (CCLE) dataset (32) (**Figure 6I**). Notably, a significant negative correlation between the NADM8 score and Inhibitory Concentration 50 (IC50) was observed, implying that a high NADM8 score might indicate potential sensitivity to the PI3K inhibitor GDC-0941 (**Figure 6J**). Furthermore, we verified the therapeutic efficacy of GDC-0941 in primary AML samples. Results suggested that patients in the NADM8^high^ group tend to be more sensitive to the treatment of PI3K inhibitor GDC-0941 as compared with the NADM8^low^ group, although it is borderline significant (P = 0.0556) due to the limited sample size (**Figure 6K**).

### Functional exploration of the core gene *SLC25A51* in the NADM8 model

**Figure 7:**
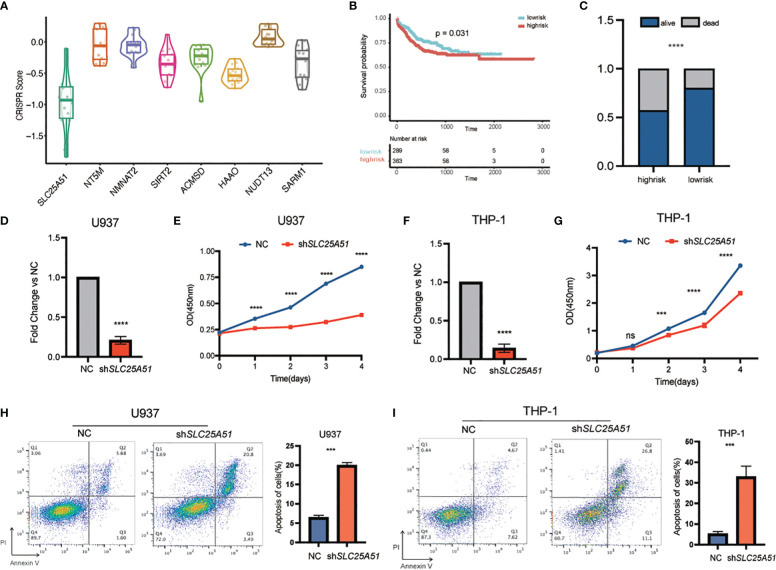
The functional exploration of *SLC25A51* in AML. **(A)** Boxplot showing the average CRISPR scores across AML cell lines of the 8 genes in the NADM8 model. **(B)** Kaplan-Meier estimates of overall survival (OS) according to the expression level of *SLC25A51* in the whole RJAML training cohort. **(C)** The overall survival status of the RJAML intermediate-risk patients between the two risk groups categorized by the median expression level of *SLC25A51*. **(D, F)** The knockdown efficiency of the *SLC25A51* gene expression level in U937 cell line **(D)** and THP-1 cell line **(F)**. **(E, G)** The proliferative capacity of U937 and THP-1 cells following stable knockdown of *SLC25A51* as measured by CCK8 assays, with red and blue lines representing the knockdown and control vector group, respectively. **(H, I)** Knockdown of *SLC25A51* could strongly induce apoptosis of U937 and THP-1 cells, as measured by annexin V/PI flow cytometric analyses. The histogram depicts the quantification of the total proportion of cells in the early (annexin V+/PI–) and late apoptotic (annexin V+/PI+) stages. All plots are representative of at least three independent experiments performed in duplicate and presented as the means ± standard deviation. (*p < 0.05, **p <0.01 and ***p < 0.001).

To further investigate the functional mechanisms of the NAD-related genes in AML, we conducted an in-depth analysis of the CRISPR-Cas9 screen dataset of 26 AML cell lines from the Dependency Map (DepMap) portal. The average CRISPR score of *SLC25A51* across AML cell lines is the lowest (-0.92) among the 8 genes in the NADM8 model, which suggests that *SLC25A51* is the most essential gene for the survival of AML cells (**Figure 7A**). *SLC25A51*, the first identified mammalian mitochondrial NAD+ transporter, has been reported to be involved in the development of various malignancies through disrupting NAD+ transport and inducing mitochondrial metabolic dysregulation (33–36). Collectively, *SLC25A51* may play a pivotal role in the pathogenesis of AML and hold great promise as a potential therapeutic target. Therefore, we selected *SLC25A51* for further exploration. The prognosis analysis showed a significant association between *SLC25A51* expression level and poor overall survival (**Figures 7B, C**). Subsequently, *in vitro* experiments were conducted to elucidate the functional role *SLC25A51* plays in the pathogenesis of AML. Two AML cell lines (U937 and THP-1) showing a high expression level of *SLC25A51* were selected for further functional assays. Following the knockdown of *SLC25A51* in cell lines, we observed a significant decrease in cell proliferation capacity (**Figures 7D–G**), as well as an increase in apoptosis (**Figures 7H, I**). Our findings underscored the pivotal oncogenic role of *SLC25A51* in the pathogenesis of AML, highlighting its potential as a promising candidate for targeted therapeutic intervention in AML.

## Discussion

Metabolic reprogramming is crucial in the pathogenesis and adaptation of tumors. The Warburg effect, which delineates abnormal aerobic glycolysis in malignant cells, is the first discovery that relates tumor biology to metabolism (37). NAD acts as a coenzyme and mediates energy metabolism and redox reactions in a wide range of metabolic pathways including glycolysis, which has been reported to facilitate the malignant proliferation of cells and play a pivotal role in many diseases (20).

AML exhibits abnormalities in numerous crucial metabolic pathways, however, there is a dearth of systematical research that links the impact of metabolic perturbations to refined risk stratification and tailored management decisions for AML patients. In this study, we substantiated the prognostic value of NAD metabolism profiles in AML, and innovatively developed a transcriptome-based prognostic model comprising eight core NAD metabolism-related genes. For instance, SLC25A51 serves as an important mammalian mitochondrial NAD+ transporter (33). SIRT2, a member of the Sirtuin family, has recently been reported as a molecular marker in predicting the poor prognosis of AML (38). NMNAT2, a nicotinamide nucleotide adenylyl-transferase, catalyzes an essential step in NAD/NADP biosynthetic pathway. Additionally, SARM1 is an important NAD hydrolase that is deeply investigated in neurodegenerative disease (39, 40).

The advantages of the established NADM8 model lie in the following aspects. Firstly, the NADM8 model was trained based on a multi-center, large-scale AML cohort, which encompassed all adult age groups, FAB subtypes, and ELN risk groups. Its prognostic value was subsequently validated in multiple independent AML cohorts. Secondly, our model exhibited superior performance compared to other transcriptome-based models in terms of both discrimination and predictive accuracy. Moreover, this simple model utilized a small number of NAD-related genes to efficiently capture the metabolic dysregulation in AML and further provided appropriate therapeutic interventions. Collectively, these findings underscore the reliability and convenience of the NADM8 prognostic model in clinical practice.

It is noteworthy that the NADM8 model can effectively distinguish a subset of AML patients with unfavorable clinical and molecular features. Patients with high NADM8 scores were older and exhibited a higher frequency of specific gene mutations in the ELN adverse risk group (e.g., *TP53*, *SF3B1*, *ASXL1*, *U2AF1*), which were reported to be enriched in elderly AML patients (41–43). Consistently, previous reports unveiled that dysregulated NAD metabolism was linked to numerous aging-related diseases (22). Moreover, the NADM8^high^ group showed higher abundance of FAB-M5 subtype across all training and validation cohorts, which may mirror potential distinctive metabolic properties associated with this specific subtype. It has been reported that monocytes rely on glutamine as a crucial energy source (44). Additionally, monocytic AML subclones exhibit resistance to BCL2 inhibitor venetoclax due to inherent molecular and metabolic characteristics (45). Such metabolic preference and dependency might provide a plausible explanation for the multidrug resistance and poor prognosis of NADM8^high^ AML patients, and further functional and mechanism experiments are warranted.

NAD metabolism is crucial for tumor microenvironment (TME) and it has been reported that NAD+ replenishment combined with PD-1/PD-L1 antibody provides a promising therapeutic strategy for immunotherapy-resistant tumors (46) and supplement of NAD+ could boost T cell-based immunotherapy (47). Analysis of the immune microenvironment yielded a surprising revelation that NADM8 high-risk patients exhibited not only an enrichment of immune cell infiltration but also a higher expression of immune checkpoint markers relative to low risk patients. Since previous studies have indicated that AML resides in an immunosuppressive state, with minimal efficacy observed in immune therapies such as immune checkpoint inhibitors. The NADM8 signature may identify patients with a unique immune state potentially yielding a higher response rate to immune therapies.

In order to deliver alternative tailored therapies on the NAD risk-based assessment, we explored *in vitro* drug sensitivity data obtained from the BeatAML dataset. Of particular note, two potential small-molecule inhibitors, the pan-PI3K inhibitor GDC-0941 and cell cycle inhibitor AT-7519, were predicted to exert therapeutic effect in NADM8^high^ patients. In line with this, the PI3K-AKT-mTOR pathway is significantly upregulated in the NADM8^high^ group through GSEA. This is one of the most frequently activated signaling pathways in cancer, which exerts a substantial influence on crucial cellular processes including cell growth, apoptosis, and metabolism, particularly glycolysis and reactive oxygen species (ROS) metabolism (48). Of note, drug sensitivity experiments on 9 AML cell lines, which showed that their sensitivity to GDC-0941 is significantly correlated with the NADM8 score, further underscored the promising potential of GDC-0941 as an effective therapeutic option for NADM8^high^ patients. To summarize, the NADM8 score could effectively characterize the specific wiring of the NAD metabolic biological process and the activation of the PI3K pathway. Patients with these features may be more susceptible to medications that interfere with this pathway, which provides a potentially effective treatment for high-risk AML patients.

Finally, we delved into the most significant gene *SLC25A51* in the NADM8 model via a series of functional studies. *In vitro* knockdown experiments demonstrated that *SLC25A51* may exert a pivotal impact on the proliferation and apoptosis of AML cells. SLC25A51 protein is localized on the mitochondrial membrane and can transport NAD+ from cytoplasm to mitochondria (33, 49). Abnormal expression of *SLC25A51* may impair the mitochondria respiratory processes, which is associated with the pathogenesis of a variety of malignancies. It has recently been discovered that SLC25A51 could sustain mitochondria acetylation homeostasis and proline biogenesis by promoting the deacetylation function of Sirtuin 3 (SIRT3), which may ultimately prompt the proliferative capacity of tumor cells. Additionally, a marked decrease of proline abundance can be detected after knockdown of *SLC25A51*, leading to the inactivation of the AKT/mTOR pathway (34). Taken together, we hypothesize that the oncogenic potential of *SLC25A51* in AML may be attributed to its capacity to transfer NAD+ into mitochondria, thereby enhancing the activity of the NAD-dependent enzymes SIRT3, SIRT4, and SIRT5 located in mitochondria, which ultimately promotes the malignant proliferation of AML cells (50). Further exploration of drugs directly target key genes in the NAD metabolism pathway may offer novel therapeutic options for high-risk patients.

## Conclusion

In summary, we established a NAD metabolism-related gene model in AML and validated its robustness and prognostic value in multiple large cohorts comprising 1870 AML patients. High NADM8 score is an independent risk factor and efficiently discriminates patients with unfavorable clinical and molecular features. Further investigations have identified potential treatment options for NADM8 high-risk patients, holding great promise to guide therapeutic decisions including both immunotherapy and targeted therapies.

## Data availability statement

The datasets presented in this study can be found in online repositories. The names of the repository/repositories and accession number(s) can be found in the article/**Supplementary Material**.

## Ethics statement

The studies involving humans were approved by Shanghai Institute of Hematology (SIH), Jiangsu Institute of Hematology (JIH), Zhejiang Institute of Hematology (ZIH). The studies were conducted in accordance with the local legislation and institutional requirements. The human samples used in this study were acquired from primarily isolated as part of your previous study for which ethical approval was obtained. Written informed consent for participation was not required from the participants or the participants’ legal guardians/next of kin in accordance with the national legislation and institutional requirements.

## Author contributions

YC: Conceptualization, Formal analysis, Investigation, Validation, Writing – original draft, Writing – review & editing. WS: Data curation, Investigation, Methodology, Writing – review & editing. PJ: Methodology, Conceptualization, Resources, Supervision, Writing – original draft. JL: Writing – review & editing, Methodology. HZ: Resources, Supervision, Writing – review & editing. XC: Resources, Supervision, Writing – review & editing. YZ: Supervision, Resources, Writing – review & editing. XH: Conceptualization, Supervision, Writing – original draft. WC: Resources, Supervision, Writing – review & editing, Writing – original draft. YS: Funding acquisition, Resources, Supervision, Writing – review & editing.

## Supplementary material

Supplementary Figure 1 NAD-related metabolism pathways are associated with poor prognosis in AML. **(A)** Forestplot demonstrates the univariate Cox survival results of the NAD metabolism-related pathways according to the GSVA score. **(B-D)** Kaplan-Meier estimates of prognostic value of the NAD metabolism-related pathways according to the GSVA score.

Supplementary Figure 2 Comparison of gene expression landscape between NADM8^high^ and NADM8^low^ patients. **(A, B)** Heatmap **(A)** and Volcano plots **(B)** show the differentially expressed genes (DEGs) between NADM8^high^ and NADM8^low^ patient groups. Significant genes were determined using the threshold of |log2(fold change) | >=1 and *P* < 0.05.

Supplementary Figure 3 Comparison of clinical information and mutational landscape between NADM8^high^ and NADM8^low^ patients in the validation cohort. **(A, B)** Waterfall plot shows the clinical information and somatic mutations between NADM8^high^ and NADM8^low^ patient groups in the BeatAML dataset **(A)** and TCGA dataset **(B)**. **(C, D)** Forest plot shows the mutations that occur at significantly different frequencies between NADM8^high^ and NADM8^low^ groups in the BeatAML dataset **(C)** and TCGA dataset **(D)**.

Supplementary Figure 4 The ex vivo drug sensitivity assays of GDC-0941 in a panel of AML cell lines. **(A-I)** The viability curves show the inhibition rate of different concentrations of GDC-0941 to a panel of AML cell lines (OCI-AML2, K562, OCI-AML3, U937, M13, KASUMI-1, HL-60, ME1, NB4) as measured by CCK8 assays. The IC50 score of GDC-0941 for each cell line was calculated by the Prism software.
