# Supplementary figures and images for: NAD metabolism-related genes provide prognostic value and potential therapeutic insights for acute myeloid leukemia
Source: Front Immunol. 2024 Jun 20;15:1417398. doi: 10.3389/fimmu.2024.1417398 (PMC11222388; doi:10.3389/fimmu.2024.1417398)

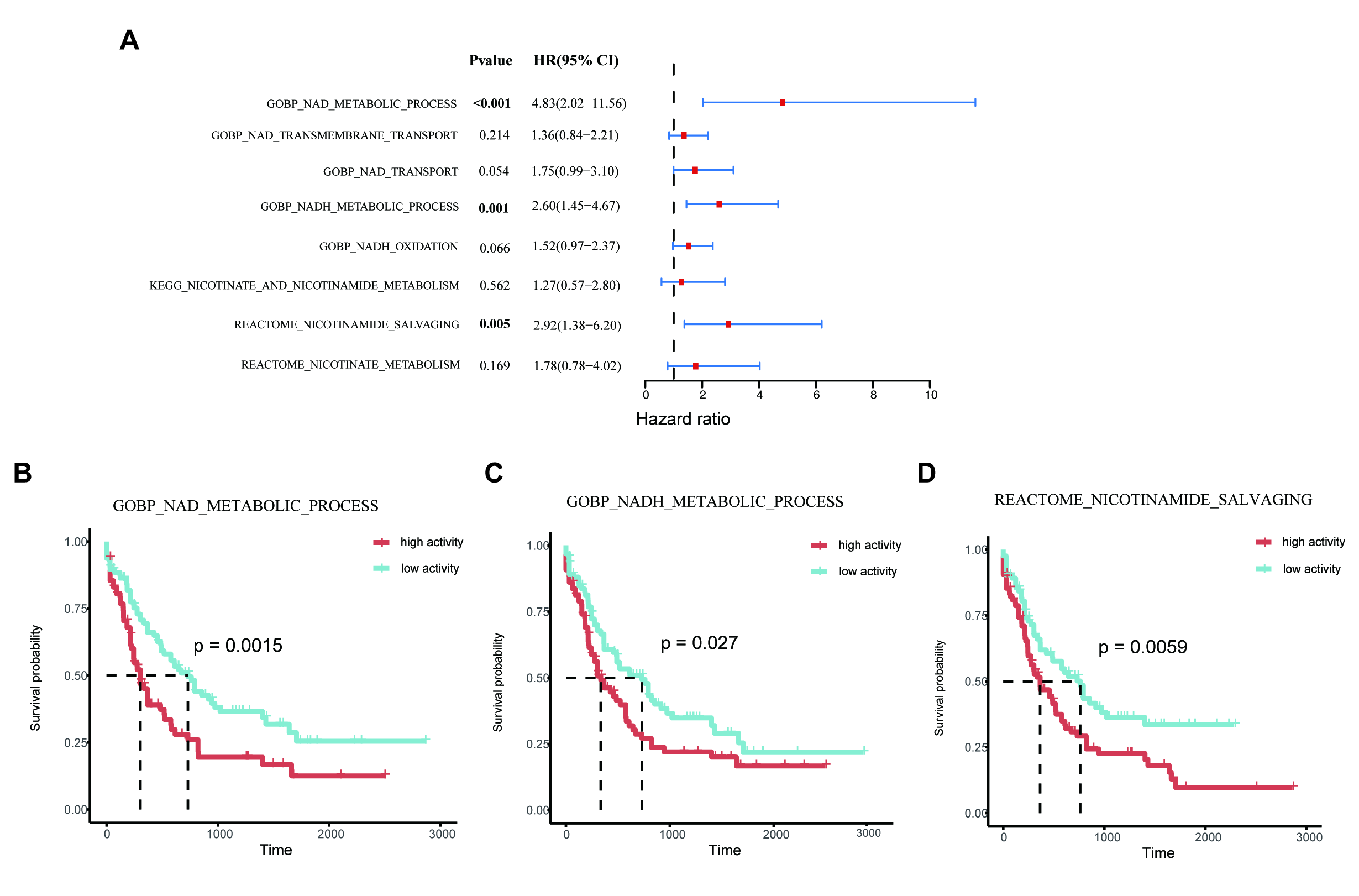

Supplement: Supplementary Figure 1 — NAD-related metabolism pathways are associated with poor prognosis in AML. (A) Forestplot demonstrates the univariate Cox survival results of the NAD metabolism-related pathways according to the GSVA score. (B-D) Kaplan-Meier estimates of prognostic value of the NAD metabolism-related pathways according to the GSVA score. [file Image_1.tif]

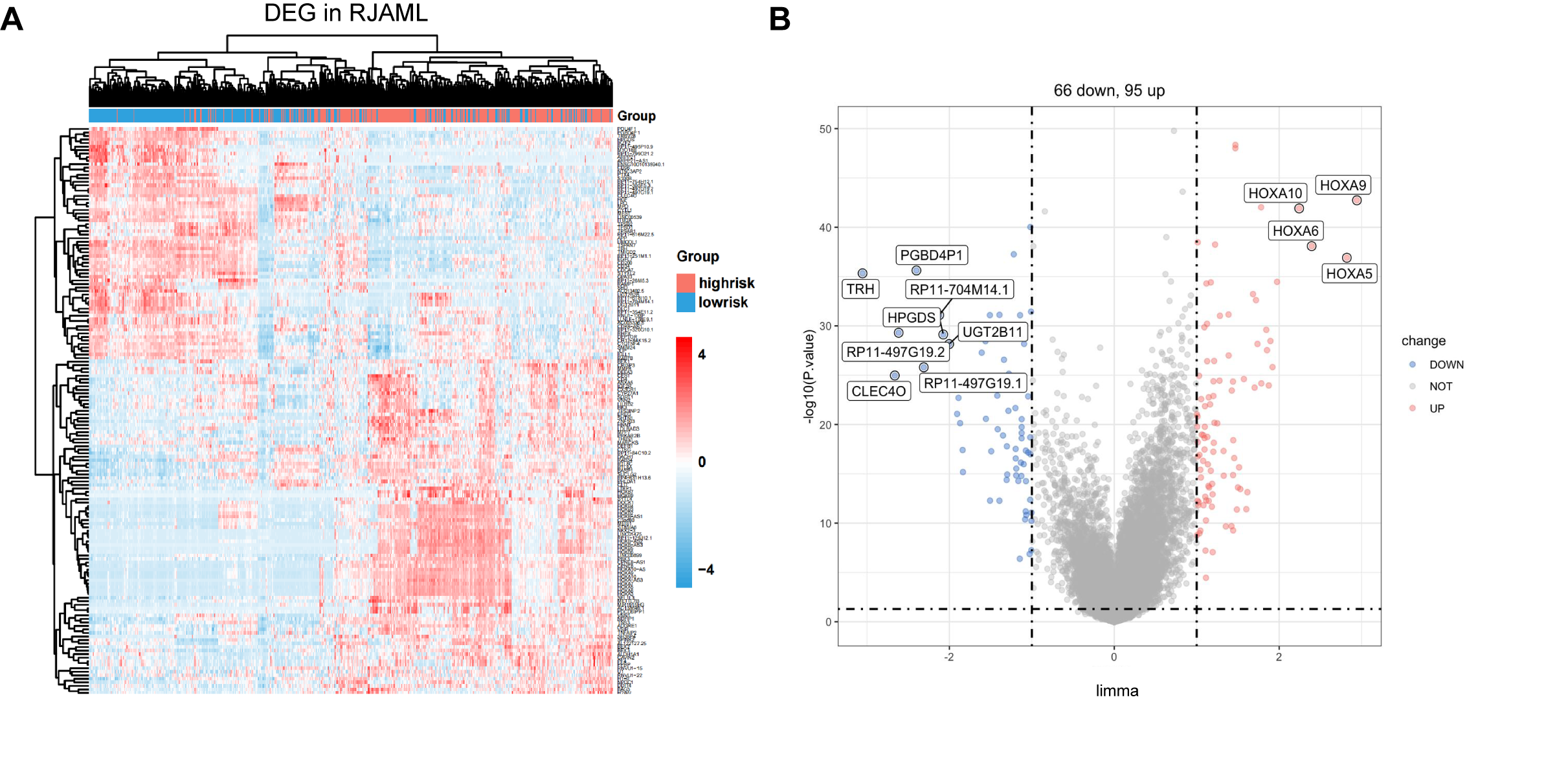

Supplement: Supplementary Figure 2 — Comparison of gene expression landscape between NADM8high and NADM8low patients. (A, B) Heatmap (A) and Volcano plots (B) show the differentially expressed genes (DEGs) between NADM8high and NADM8low patient groups. Significant genes were determined using the threshold of |log2(fold change) | >=1 and P < 0.05. [file Image_2.tif]

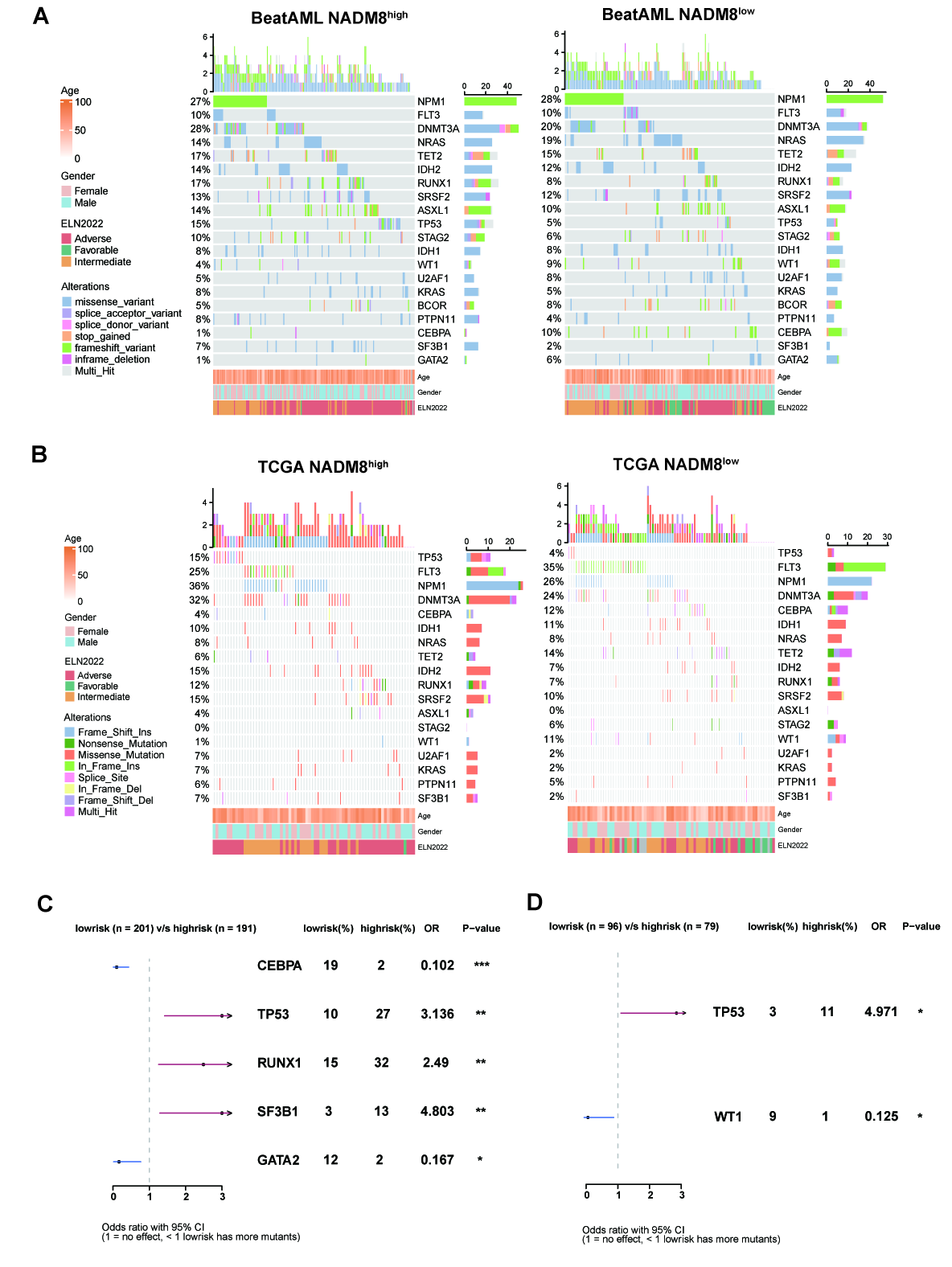

Supplement: Supplementary Figure 3 — Comparison of clinical information and mutational landscape between NADM8high and NADM8low patients in the validation cohort. (A, B) Waterfall plot shows the clinical information and somatic mutations between NADM8high and NADM8low patient groups in the BeatAML dataset (A) and TCGA dataset (B). (C, D) Forest plot shows the mutations that occur at significantly different frequencies between NADM8high and NADM8low groups in the BeatAML dataset (C) and TCGA dataset (D). [file Image_3.tif]

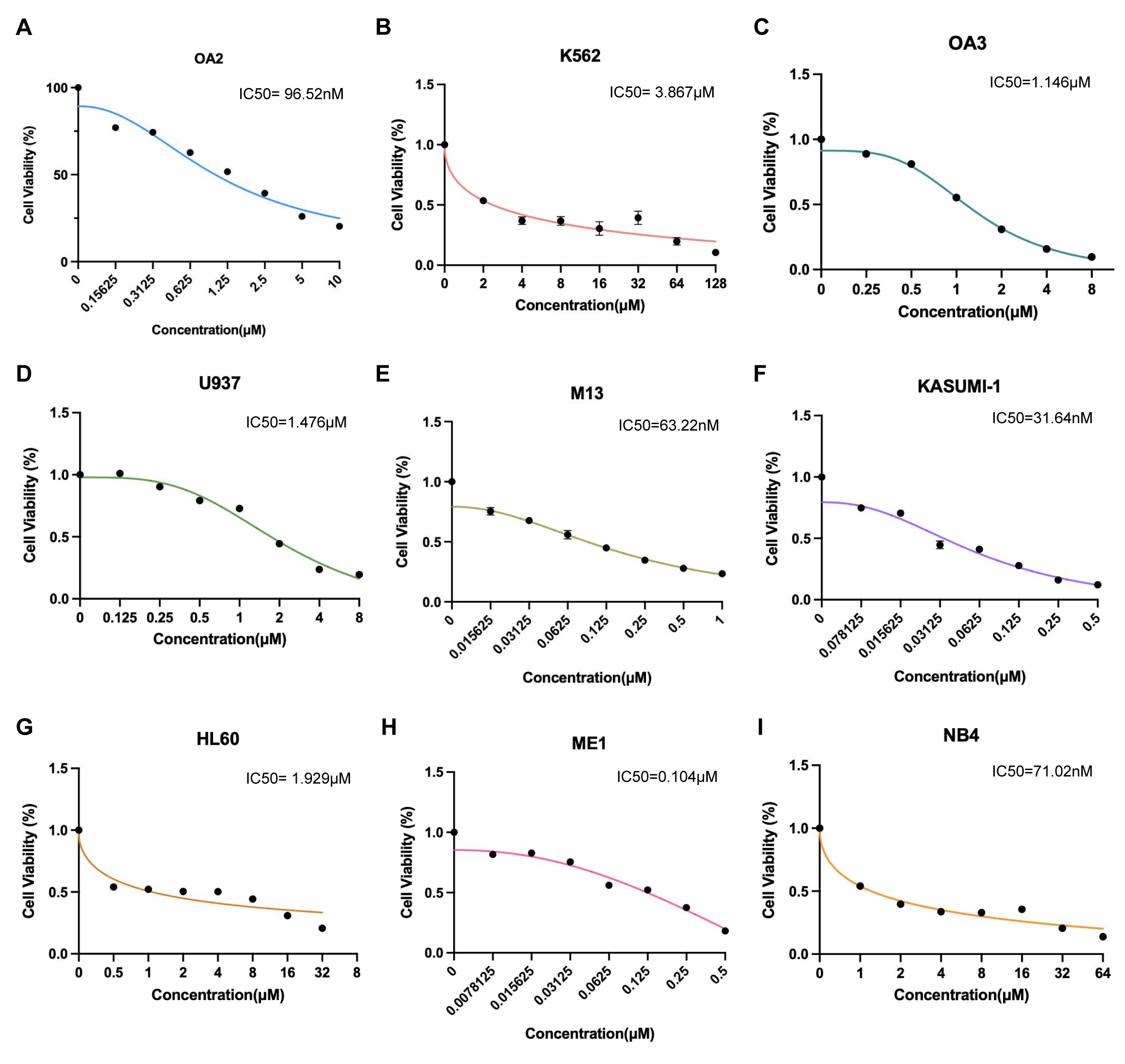

Supplement: Supplementary Figure 4 — The ex vivo drug sensitivity assays of GDC-0941 in a panel of AML cell lines. (A-I) The viability curves show the inhibition rate of different concentrations of GDC-0941 to a panel of AML cell lines (OCI-AML2, K562, OCI-AML3, U937, M13, KASUMI-1, HL-60, ME1, NB4) as measured by CCK8 assays. The IC50 score of GDC-0941 for each cell line was calculated by the Prism software. [file Image_4.tif]
