# Supplementary material for: NAD metabolism-related genes provide prognostic value and potential therapeutic insights for acute myeloid leukemia
Source: Front Immunol. 2024 Jun 20;15:1417398. doi: 10.3389/fimmu.2024.1417398 (PMC11222388; doi:10.3389/fimmu.2024.1417398)
Supplement: Supplementary file 5 [file DataSheet_1.docx]

**Materials and Methods**

**Data availability and treatment**

The development cohort comprised 655 newly diagnosed AML patients from three large centers, 442 of these patients were from Shanghai Institute of Hematology (SIH), 110 from Jiangsu Institute of Hematology (JIH), and 103 from Zhejiang Institute of Hematology (ZIH). All bone marrow (BM) samples from 655 AML patients were collected at diagnosis. Anonymized [RNA sequencing] data have been deposited in [The Genome Sequence Archive for Human (GSA-Human, https://ngdc.cncb.ac.cn/gsa-human)] (HRA002693). All Bone marrow (BM) samples were collected at diagnosis. This study was approved by the three participating centers. All patients had given informed consent for both treatment and cryopreservation of BM and peripheral blood samples according to the Declaration of Helsinki.

For non-M3 AML, young patients (< 60 years) were given standard intensive “3+7” IA-based regimens as initial induction, and after CR was achieved, 4 cycles of high-dose cytarabine were delivered as consolidation. Elderly patients (≥60 years) were evaluated by the treating physician. Fit patients received reduced IA/DA-based induction chemotherapy, while unfit patients were assigned to other less intensive therapies. For acute promyelocytic leukemia (APL) patients, the combination of All-trans retinoic acid (ATRA) and Arsenic trioxide (ATO) with or without chemotherapy was administered based on Sanz risk stratification. The diagnosis and subtype classification of AML were based on the 2022 World Health Organization criteria.

The validation cohorts were obtained from TCGA (https://portal.gdc.cancer.gov/), BeatAML (http://www.vizome.org/), and HOVON cohort retrieved from Array Express (Dataset ID: E-MTAB-3444). Only treatment-naive adult patients were included, and individuals without available survival information were excluded.

**Acquisition of NAD metabolism-related genesets**

The NAD metabolism-related genes were obtained from the Molecular Signatures Database (MSigDB), including REACTOME_NICOTINATE_METABOLISM(R-HAS-196807) from Reactome database, KEGG_NICOTINATE_AND_NICOTINAMIDE_METABOLISM (has00760) from KEGG database, and GOBP_DE_NOVO_NAD_BIOSYNTHETIC_PROCESS, GOBP_NAD_TRANSPORT from GO database. After integrating the genes in these pathways, a total of seventy-seven genes were selected. Detailed information about the NAD+ metabolism-related genes is contained in (Supplementary Table S1).

**Developing and validation of AML prognostic model**

The least absolute shrinkage selector operator (LASSO) regression algorithm was employed in the *glmnet* R package to select the fitness genes that could be utilized to develop a predictive model, ultimately generating eight genes most pertinent to prognosis. The seed number was “1006” and the “lambda.min” was selected for the model. Then, the 8 genes were incorporated into the multivariate Cox regression and established the NADM8 model. The NADM8 risk score was calculated using the formula: NADM8 score = Σ (β*i* × expression level of gene*i*), where β*i* was the coefficient generated by multivariable Cox regression analysis. Furthermore, each patient was allocated a risk score according to the formula and subsequently divided into high-risk and low-risk subgroups according to the median cut-off risk score. Kaplan-Meier survival analysis was employed to assess the prognostic implications of the NADM8 model using the *survival* and *survminer* R package. The time-dependent ROC curves and area under time-dependent ROC curve (AUC) were performed using the *timeROC* package.

**Gene set variation analysis (GSVA) and pathway survival analysis**

We systematically collected the NAD metabolism-related pathways from the "msigdb.v2023.1.Hs.symbols.gmt" in MSigDB database(1) and conducted Gene Set Variation Analysis (GSVA) utilizing the *GSVA* R package to calculate a score of each pathway for the patients. Then, survival analysis was performed using univariate Cox regression analysis and Kaplan-Meier plotter to explore the prognostic value of these pathways. *P* value<0.05 was determined as significant.

**Differential gene expression analysis and gene set enrichment analysis (GSEA)**

The differential expression analysis was conducted by the *limma* R package comparing the raw count data between NADM8^high^ and NADM8^low^ patients. Differentially expressed genes were determined with the threshold of |log_2_(fold change) | >=1 and *P* < 0.05. All the genes ordered in rank were used as input for GSEA analysis utilizing the *clusterProfiler* R package. Hallmark genesets "h.all.v2023.1.Hs.symbols.gmt", KEGG pathway "c2.cp.kegg.v2023.1.Hs.symbols.gmt", Reactome geneset "c2.cp.reactome.v2023.1.Hs.symbols.gmt", and GO geneset "c5.all.v2023.2.Hs.symbols.gmt" from MSigdb were used for GSEA analysis. *P* value<0.05 was regarded as significant.

**Cell culture**

All the human AML cells were obtained from the Shanghai Institute of Hematology. NB4, HL-60, MV-411, MOLM13, U937, OCI-AML3, and SKNO-1 were cultured in RPMI 1640 medium (BasalMedia, L210KJ) supplemented with 10% fetal bovine serum (FBS), 1× penicillin-streptomycin and 2 mM L-glutamine. Kasumi-1 was cultured in RPMI 1640 (BasalMedia, L210KJ) medium supplemented with 20% fetal bovine serum, 1× penicillin-streptomycin. OCI-AML2 was cultured in α-MEM medium (BasalMedia, L570KJ) supplemented with 20% fetal bovine serum and 1× penicillin–streptomycin. The cells were maintained in a humidified incubator at 37°C with 5% CO2.

**Drug sensitivity assays**

A panel of AML cell lines (HL-60, U937, OCI-AML3, Kasumi-1, ME1, NB4, MOLM13, OCI-AML2, K562) were exposed to varying concentrations of GDC-0941 (Selleckchem, S2065) in 96-well plates at a density of 2 × 10^4^ cells per well. The stock solutions of GDC-0941 were dissolved in dimethyl sulfoxide to a 10mM. After incubation for 48h, cell viability was assessed using the Cell Counting Kit-8 (CCK-8) (Dojindo). 10ul CCK-8 solution was added to 96 well plates with the cells and further incubated at 37 ℃, 5% CO2 for 4 h. Then, the absorbance of the samples was measured at 450 nm. The RNA-Seq data of AML cell lines were downloaded from the Cancer Cell Line Encyclopedia (CCLE) (https://sites.broadinstitute.org/ccle/datasets) and the risk scores were calculated according to the NAGM8 equation. Pearson's regression analysis was conducted to examine the correlation between risk scores and IC50 values. *P* value <0.05 was regarded as statistically significant.

**Functional experiments in AML cell lines**

To achieve knockdown of the *SLC25A51* gene, we constructed a pLVX plasmid carrying shRNA (shRNA sequences are: TRCN0000230321, Forward sequence: GATCCGCATGGAATTGGAGAGTATTATTTCAAGAGAGTACCTTAACCTCTCATAATATTTTTTG, Reverse sequence: AATTCAAAAAACATGGAATTGGAGAGTATTATTCTCTTGAAGTACCTTAACCTCTCATAATACG). The pLVX plasmid (with GFP), along with psPAX2 and pMD2.G plasmids, were co-transfected into HEK293T cells using lipofectamine 2000 (Invitrogen, 11668027). The supernatant was collected at 24h and 48h post-transfection to obtain the viral particles. To generate stable knockdown cell lines, the THP-1 and U937 cells were seeded at 2 × 10^5 cells/well in 6-well plates and infected with 2ml lentiviral supernatant in the presence of 8 μg/ul polybrene (Sigma-Aldrich, TR-1003) at 1200g for 90 minutes , at 30°C–32°C. After 48 hours, GFP positivity was assessed by flow cytometry to determine transduction efficiency. RNA and protein were extracted to evaluate the knockdown efficiency through quantitative methods. At 72 hours post-transduction, flow cytometry was employed to assess the apoptosis (Invitrogen™ eBioscience™ Annexin V Apoptosis Detection Kits) and cell cycle distribution (Cell Cycle Staining Kit, Multi Science). Cell proliferation was monitored for four consecutive days using the CCK-8 assay (Cell Counting Kit-8, DOJINDO). Cells were seeded into 96-well plates at 2000 cells/well, and at 0, 1, 2, 3 and 4 days, CCK-8 was added to each well and incubated at 37 °C for 4h. Then the optical densities (ODs) were measured at 450 nm.

**RNA extraction and Quantitative Real-Time PCR (qRT-PCR)**

Total RNA was isolated from AML cell lines using the EZ-press RNA Purification Kit (*EZ*bioscience) and then reversely transcribed into complementary DNA (cDNA) according to the manufacturer's protocol using a HiScript® III RT SuperMix for qPCR (+gDNA wiper) (Vazyme). qRT-PCR was performed with the ChamQ Universal SYBR qPCR Master Mix (Vazyme). The primers used for qRT-PCR are as follows: SLC25A51: Forward sequence, 5’-GGACGCCGGCGCTGAT-3’, Reverse sequence, 5’-GCTTCTGAATCCATCATGCTGCT-3’; GAPDH: Forward sequence, 5’-GAAGGTGAAGGTCGGAGTC-3’; Reverse sequence, 5’-GAAGATGGTGATGGGATTTC-3’. GAPDH was used as the internal control.

**Statistical analyses**

The statistical analyses in this study were conducted by R 4.3.1. For the statistical significance estimation of clinical characteristics and outcome, the Student’s t-test was applied for normally distributed quantitative data, the Wilcoxon test for non-normally distributed continuous variables, and the Fisher's exact test was utilized for categorical data. The Cochran-Mantel-Haenszel (CMH) test was employed to assess unidirectional ordered contingency tables. The mutational profile was analyzed via the *maftools* package. The mutation waterfall plot was drawn using the R package *complexheatmap*. The influence of specific mutation on AML patient survival was evaluated with K-M plot. Utilizing the *survival*, *regplot* and *rms* R packages, we constructed a nomogram and generated the calibration curve. The GO and KEGG analysis were performed by R package “clusterProfler” with the reference gene sets from "c5.all.v2023.2.Hs.symbols.gmt" and "c2.cp.kegg.v7.5.1.symbols" which were downloaded from MSigDB.

1. Liberzon A, Birger C, Thorvaldsdóttir H, Ghandi M, Mesirov JP, Tamayo P. The Molecular Signatures Database (MSigDB) hallmark gene set collection. Cell Syst. 2015;1(6):417-25.
